# “Son of the Soil … Daughters of the Land”: poetry writing as a strategy of citizen-making for lesbian, gay, and bisexual migrants and asylum seekers in Johannesburg

**DOI:** 10.1080/10130950.2016.1189652

**Published:** 2016-07-01

**Authors:** LeConté J. Dill, Jo Vearey, Elsa Oliveira, Gabriela Martínez Castillo

**Keywords:** poetry, LGBTIQ, migration, citizenship, participatory methodologies, South Africa

## Abstract

South Africa’s Constitution, Bill of Rights, and Freedom Charter are globally ground-breaking for providing provisions of non-discrimination, and, of particular note, on the basis of sexual orientation. Since the introduction of these protective frameworks, lesbian, gay, bisexual, transgender, intersex, and queer (LGBTIQ) communities, allies, and advocates in the country have won major legal battles on these issues; however, in spite of these successes, LGBTIQ communities continue to face hostility and violence. As a result, South African LGBTIQ individuals often travel to urban centres, such as Johannesburg, in the hope that these spaces will be more tolerant of their sexual orientation and gender identity; the reality, however, suggests otherwise. Moreover, despite South Africa’s designation as a safe haven for LGBTIQ communities, migrants from other African countries — where same-sex relationships are criminalised — are overwhelmingly met with xenophobic verbal, emotional, physical, and political violence. This article describes the authors’ engagement with nine lesbian, gay and bisexual (LGB) migrants and asylum seekers from Zimbabwe, Malawi, and elsewhere in South Africa during a weeklong poetry workshop exploring their lived experiences in Johannesburg. This workshop followed a body mapping and narrative writing workshop held previously with the same participants. This article investigates the themes identified from the body mapping process that guided the poems produced: migration, violence, citizenship, and freedom. The poetry created during the workshop illuminates how lesbian, gay, and bisexual migrants in Johannesburg work on a daily basis to build social trust as they demand to be seen and recognised, to enact their rights, to make and remake homes, to show up in public as Black people, as LGB individuals, and as human beings. We explore these strategies of citizen-making as informed by the LGB poets with whom we had the opportunity to work.

## Introduction

South Africa’s Constitution, Bill of Rights, and Freedom Charter are globally ground-breaking for providing provisions of non-discrimination. Of particular note, the Equality Clause in Section 9 of the Constitution (Republic of South Africa, 1996) protects freedom, equality, and dignity on the basis of sexual orientation, among other identities. Coupled with legal victories during the last 20 years, these laws are an attempt to address the increasing inequalities that plague the country (Nath, 2011). As a result of its progressive legal framework, South Africa has been sought out as a safe haven for lesbian, gay, and bisexual (LGB)^1^ individuals from across the continent, where same-sex relationships are criminalised. South Africa’s Refugee Act, first drafted in 1998, “at a time when the South African commitment to rights was strengthened by the memory of the abuses of apartheid” (MoVE, 2016:16) incorporates progressive provisions for individuals to apply for asylum on the basis of violence and/or, persecution, or fear of such, associated with their sexual orientation and gender identity. However, although the Refugee Act no 130 of 1998 technically provides an opportunity of safety for individuals who experience criminalisation and discrimination in their countries of origin, in reality, the implementation of this progressive legislation is fraught with inconsistencies and weak implementation. Research suggests that a successful application on the basis of sexual orientation and/or gender identity is unlikely (Middleton, 2009; Palmary, 2009; Palmary *et al*, 2010). Reasons for this include “corruption to inaccessibility of the system to appallingly poor decision making by the Refugee Status Determination Officers” (MoVE, 2016:16).

The state is obligated to perform a certain way towards its citizenry (Marshall, 1950). However, according to the notion of “biopower,” the state also can use its authority to choose to identify or to neglect certain individuals and populations, or in the words of Foucault, to both “make live” but also “let die” (1978:137; Pagano, 2011). This practice is the foundation upon which inequality can be built (Creary, 2016). In the context of South Africa, the bodies of LGB migrants and asylum seekers there, and the mobility of these bodies, are constantly surveilled and controlled by the state under the frame of exerting its biopower. In conjunction with the increasingly restrictive Immigration Regulations of 2014 that make it difficult, if not impossible, for individuals without critical skills to regularise their long-term stay in South Africa, many non-national LGB migrants struggle to obtain and maintain a legal documentation status.^2^ Therefore biopower promotes a “narrative of contagion” and, in this case, marks the bodies of migrants and asylum seekers in South Africa as problematic, risky, deviant, or dangerous (Mitropoulos, 2012:46).

In spite of the protective legislation in place, LGB individuals who are also migrants to South Africa, face multiple forms of both structural and direct violence that threaten their lived experiences, including xenophobia and anti-foreigner sentiments, verbal and physical abuse, challenges in accessing documentation, basic social services and justice, and a lack of substantive citizenship. Concomitantly, heteronormative pressures of conformity prevalent in the region also impact the lives of those who are sexual and gender non-conforming. Fears and experiences of physical, sexual and emotional violence, bullying and harassment, blackmail, and other oppressive practices substantially influence the ways and degree to which LGB persons are safely and freely able to express themselves (Marnell and Khan, 2016).

In order to gain increased insights into the lives of LGB migrants we argue that it is necessary to involve those who are experiencing the issues under investigation in the research process. Influenced by the work of Paulo Freire, Cornwall and Jewkes (1995:1674) argue that participatory research is about “empowering disenfranchised and marginalized groups to take action to transform their lives” where knowledge is gained by using a “bottom-up” approach that focuses on a process of “sequential reflection and action carried out *with* and *by* local people rather than *on* them” (1995:1667). Participatory arts-based methods, such as those applied in the projects that inform this article, encompass a range of strategies and approaches meant to facilitate participant-centred “meaning-making” through collaborative processes as individual participants and/or as a participatory group (Blackbeard and Lindegger, 2015:87). The artifact(s) that are created by the participants can “serve *as* data, and may also *represent* data” (Leavy, 2009:227). Mitchell (2011) argues that visual research can be used to educate people and empower communities where the interpretive process is not dependent solely on what the researcher and participant believe to be true but that the audience is also afforded an ability to engage in a process whereby they reflect on what the creator might be saying and/or revealing. Critics of arts-based methods question the nature of subjectivity of representation, “but it is exactly this subjectivity that can unlock insights into the complexities and intersections of experiences” (Oliveira and Vearey, 2015a:314).

The title of this article references a poem by South African *imbongi* (praise poet) Zolani Mkiva (n.d.) entitled ‘Son of Soil’ in which he shares:

I am the son of the soil

Like daughters of the land

I am the filament of freedom

I am the pistil of peace

I am the calyx of consciousness

I am the corolla of peoples cause

I am the pollen of prosperity

I am the anther of amicable solutions

I am the stem of our society

The son of the soil

Our paper is informed by “son[s] of the soil” and “daughters of the land” - LGB migrants in Johannesburg who share their experiences with internal and cross-border displacement, as well as their varied strategies of place-making in South Africa.

## Method

This article draws on work undertaken as part of the MoVE:method:visual:explore project^3^, coordinated by the second and third author, based at the African Centre for Migration & Society (ACMS) at the University of the Witwatersrand. MoVE focuses on the development of visual and other involved arts-based methodologies, in conjunction with more traditional qualitative research methods, to research the lived experiences of migrants in southern Africa (MoVE:method:visual:explore, 2016). This approach aims to integrate social action with research, and involves collaboration with migrant participants, existing social movements, qualified facilitators and trainers, and research students engaged in participatory research methods. These approaches involve the co-production of knowledge through the development of partnerships with under-represented migrant groups (Vearey *et al*, 2011; Oliveira and Vearey, 2015a; Oliveira and Vearey, 2015b; Oliveira and Vearey, 2016).

In November 2015, the authors engaged with nine LGB adult migrants and refugees from Zimbabwe, Malawi, and elsewhere in South Africa in a week-long poetry workshop in Johannesburg. The workshop involved nine of the eleven participants from the *Queer Crossings* Project, a seven-day arts-based research workshop led by the third author, in collaboration with the Gay and Lesbian Memory in Action (GALA) project and Seattle University, which began in 2014. Using a visual arts methodology (body mapping) and narrative writing, the project explored the lived experiences of LGB migrants, asylum seekers, and refugees living in Johannesburg (MoVE, 2016). According to Gabriel Hoosain Khan, a facilitator from GALA, the body maps and narratives:

reflect the fragmented, multiple, and intersecting realities of the participants’ lives. The images and narratives produced by the participants/artists foreground the complex and diverse narratives of LGB asylum seekers and migrants. This body of work can challenge the academy to rethink what constitutes valid knowledge and who has the opportunity to be heard in the process of knowledge production (MoVE, 2016:116).

Prior to the November 2015 workshop, contact was made with the original *Queer Crossings* participants via their cell phones through the mobile messaging application *WhatsApp*. The idea of the poetry workshop was shared and individuals were invited to participate. Of the original eleven participants, nine joined the poetry workshop. Some travelled from as far as Zimbabwe to be able to participate. Workshop participants and the authors co-created a collective safe space in which to read, write, and share poems. Soon after the beginning of the workshop, the participants began to refer to themselves as “poets,” thereby claiming this identity as a “performance of possibilities” (Madison, 2005; Winn and Jackson, 2011).

All travel costs were reimbursed and a daily transport stipend of ZAR 100 (USD 6; sufficient for a daily return journey to and from the workshop venue) was provided in addition to refreshments throughout the workshop and lunch each day. After an information session held at the start of the workshop, all poets gave consent to participate and for the poems of their choosing to be made publicly available online and in print, and for them to be used in other associated publications, such as this article. On the final day of the workshop, poets gave a reading of their work at the Women’s Gaol at Constitution Hill in Johannesburg; a fitting site to share stories of struggle. This was followed by a tour of the Constitutional Court and a celebratory lunch. Each poet invited two guests to the celebration for whom transport costs were reimbursed, and a printed chapbook of the poems was produced.

The November 2015 workshop was organised around four themes identified from an analysis of the body maps, narrative writing and reflections produced in the 2014 workshop — migration, violence, citizenship, and freedom. During the November 2015 workshop, the poets reflected upon and discussed how these themes impact their lives, read poems from published poets across the pan-African diaspora, and wrote “interpretive poems” (Langer and Furman, 2004:8) based on the themes and writing prompts developed through the method of “participatory narrative analysis” (Dill, 2015). The findings below refer to the authors’ analysis of the poetry produced by the participants. The poetry workshop was co-facilitated by the first author and South African poet Makhosazana Xaba, a Writing and Documentation Fellow at GALA, and field notes were taken during and after the workshop by all of the authors. The poets named the workshop “#weareallpoets”^4^. Ethics approval for the study was granted by the University of the Witwatersrand Research Ethics Committee (non-medical).

## Findings

### Participants

The nine poets were between 25 and 32 years of age. The seven non-nationals - reflecting the challenges of South Africa’s increasingly restrictive Immigration Act - held different immigration documents, from asylum seeker permits, to visitor permits, to being currently undocumented. Two of the nine poets - Babymez^5^ (32 years old) and Modise (29 years old) - originated from elsewhere in South Africa, having travelled to Johannesburg from KwaZulu-Natal in 2000 and the Eastern Cape in 2011, respectively. Hotstix (25 years old), Jonso (age unknown), Marlon (28 years old), Petunia (27 years old), Shane (29 years old), and Tino (30 years old) are Zimbabwean nationals who have been in Johannesburg for between two and ten years. One poet - Timzy (28 years old) - travelled to Johannesburg from Malawi in 2007.

### Migration

We began the weeklong poetry workshop by discussing the theme of migration. The facilitators shared Keorapetse Kgositsile’s poem ‘In the naming’ (Kgositsile, 2004) with the group, in which he describes places as being inviting or uninviting, but moreover that “places can have scars” (Kgositsile, 2004:34). One of the initial writing prompts that we gave the poets was to write an “I Come From” poem describing the places, spaces, cultures, and identities in which they find alignment. Marlon, a lesbian woman from Zimbabwe, shared the following:

I come from still waters to flowing rivers

I come from cold winters to hot summers

I come from dry desserts to wet forests

I come from closed closets to open closets

I come from rainy nights and woke up to rainbows

I come from shackles that bind to shackles broken

I come from dead silence to words spoken

I come from paths unknown to paths chosen

In ‘I Come From’, Marlon begins to explore the dichotomy between places and identities that are restricted to ones that are more welcoming, and begins to allude to her migration from being lesbian in Zimbabwe to migrating and being an out lesbian in South Africa.

The poets revisited their body maps created in 2014, and developed Ekphrastic poems - poems describing a work of visual art - based on them. Hotstix, also from Zimbabwe, wrote the following in an excerpt from the Ekphrastic poem entitled ‘My Body Work’:

I am human

I am any image of God

Coming from my country was not easy

Not because they chased me away but my soul needed to

I came from a place where no baboons would want to stay

I am a lesbian and I’m proud

In this poem, Hotstix also describes an unwelcoming homeland and an emotional need to migrate to a place where she can be openly lesbian.

### Violence

On the second day of our poetry workshop, we transitioned to discussing violence, particularly as the migration process stems from experiences of violence and/or migrants still experience various forms of violence in their new destinations. Specifically, the poets shared experiences of xenophobic violence and homophobic violence, both verbal and physical. In an excerpt from an Ekphrastic poem based on her body map, Modise, originally from the Eastern Cape (South Africa), shares the following:

Am me and am happy to be me

The world went rough on me but I didn’t change of being me. They call names, bitch, slight whore, isitabane

But I kept going and stronger day by day

Who are you to tell me what I am

If you don’t like what you see, Phuma Kimi!

In this excerpt, Modise describes the verbal abuse enacted by being called derogatory names for women and lesbians. At the end of the poem, she offers her own strong retort, meaning “get out of my business.” The poets also described verbal and physical abuse due to their intersectional histories and identities as sort of an erasure of the self. Consequently, the group wrote Erasure poems^6^, whereby you erase words from an existing text, using the leftover words to form a poem. Taking South African Afropop singer Brenda Fassie’s song ‘Too late for Mama’ (Fassie, 1989), Hotstix wrote the following Erasure poem entitled ‘She is Gone’:

Ten kilometers barefooted the bush

Started raining on the way

She tried hiding under a tree to save her child

Lightning caught her

Friends, relatives ran for her

it was too late

Husband came running

Poor man held his dead wife

Knelt down and prayed for this

It was too late

She is gone

In this Erasure poem, Hotstix recounts a narrative of poverty, strategies of survival, but ultimately a violent death.

### Citizenship

On the third day of the poetry workshop, we discussed the theme of citizenship, as the previous days’ discussion began to highlight the limited recognition and rights that they have being Black, migrants, and LGB. In this excerpt from ‘Black Maid,’ Hotstix continues the themes from ‘She is Gone,’ reflecting on the multiple burdens that Black women, with liminal ‘citizenship,’ must endure. She shares:

She needs to be loved and to make love sometimes

But how? Master need a clean ironed suit

Listening to those kids noise kills her

She left hers, her heart pounds with anger

She is supposed to take care of cats

Yet her grandchild is dying

She is black and not free

She is black and she should die working

She is black and strong,

She is my Granny

In describing the liminal citizenships of migrants, in ‘A Cry of a Kwere-kwere’, Shane, a gay man from Zimbabwe, shares:

My Silence doesn’t mean am a fool

I might Be a Kwere-Kwere but still a Human being

Treat me with Respect.

“Kwere-Kwere,” said to be an onomatopoeic term for how migrants and immigrants sound to South Africans, becomes a derogatory term for “foreigners” like Shane.

Tino, also a gay man from Zimbabwe, continues to describe this liminal citizenship, particularly for someone with multiple marginalised identities, being Black, a migrant, and gay. Leaning on Kgositsile’s poem and our article’s title, in an excerpt from ‘Son of Soil,’ Tino shares:

Taboo, unnatural, outcast, became,

Lesson learnt at least now

You know, a shame, disgusting, evil

I brought, both to the family and

to the nation.

Here I am the son of the

Soil, never learnt, read, borrow

or inherit it, I am convinced I am

not one, we a lot, only that you

are hiding or denying about

It’s unAfrican western thing,

I never attended a white school

Or never been with them, I am

Pure African-Zimbabwean, never been

Out of African-I am a real black

faggot.

### Freedom

On the fourth day of the poetry workshop, we discussed and interrogated the theme of freedom. Many of the poets wrote Praise poems - an African traditional form often in which elders remind the youth who they are, that they belong, that they are loved, and that they each have special gifts. In an excerpt from ‘Mama Africa,’ Timzy, a gay man from Malawi, shares:

Oh mama Africa

It’s been long Since we Children of Africa

We have been crying for freedom

We don’t even knows where we belong anymore

OH MAMA AFRICA

We don’t have a place to call home anymore

Since we run away from our original homes

To the foreign country

OH MAMA AFRICA

We are one we belong to the same place

Let’s put our weapons down

In imagining freedom, Timzy repeats the direct address to “Mama Africa” and speaks of migration, violence, and the lack of citizenship with which marginalised populations must contend. At the end of the poem, he reminds us that “we are one.” “Mama Africa” is also South African singer and activist, Miriam Makeba’s moniker. In an Erasure poem of her song ‘Thulasizwe/I Shall Be Released’ (Makeba, 1969), Jonso, from Zimbabwe, shares:

Oh any day now

I swear

I shall be released

As the poets explore this pursuit of freedom, Marlon expounds on this process:

As I step into the light, my face revealed

I am in motion, moving, I am proceeding

### Citizen-making as a constant pursuit

In March 2016, a public exhibition and publication launch that included the body maps produced by the participants alongside their narrative stories and poems was showcased at the Workers’ Museum in Newtown - an inner-city area of Johannesburg.^7^ With the exception of two poetry workshop participants who could not attend due to work commitments, all workshop participants were in attendance at the launch event. Many of them read their poems and discussed their visual and narrative works with the exhibition attendees.

The exhibition ran for two months and has received positive feedback from the general public.^8^ According to Beltran and Begun (2014), the process of testimony and public witness to experiences can be transformative for participants. The participants who were in attendance expressed pride in their work and in the importance of sharing their stories with public audiences. Tino stated during the exhibition,

“People need to be exposed to our stories as often as possible. In order for our lives to improve we need the public to understand the issues and challenges that we face. Maybe this way the Department of Home Affairs will change and do the right thing.”

The re-telling of life stories in an artistic form can legitimise the experience of the participant, and according to O’Neil (2002:75), “outcomes of participatory research can inform, educate, remind, challenge and empower both those involved and the audiences of the research outcomes.” In this vein, Babymez highlighted how the project provided her with a level of healing during an informal interview with the third author held after the exhibition:

“The workshops were amazing and I learned so much about myself from them. When I was creating my body map I had no idea that I would visit painful stories in my life but through that work and through the writing and the poems I have been able to heal some of those wounds and this is so important for me as a person. Seeing the exhibition up makes me feel so strong and proud to be who I am.”

The exhibition and publication of *Queer Crossings* was the first time that LGB migrants and asylum seekers in South Africa had shared their stories and memories in print and with the public. According to Anthony Manion, the Director of GALA at the time of the projects:

the works powerfully convey the experiences of LGB migrants and asylum seekers who had hoped that Johannesburg would be a safe haven and instead have met serious challenges in terms of safety and access to services. In many cases the contributors to this anthology share stories that they had kept hidden out of fear that they would be victimised if their sexuality or gender identity were discovered.

He goes further by saying, “It is hoped that the stories contained in this anthology will contribute to a better understanding of the experiences of this marginalised group and stimulate further discussion, research and activism” (MoVE, 2016:12).

## Discussion

Poet, politician, and founder of the Negritude Movement, Aimé Césaire said that “poetic knowledge” is the notion that poetry creates the kind of knowledge needed to transform oppressive conditions (Cesaire, 1945:158; Kelley, 2002:9). The poems produced in the #weareallpoets workshop provide some knowledge and insights into the lived experiences and strategies of citizenship-making among LGB migrants and asylum seekers in Johannesburg.

Like many migrants, the poets in our workshop moved for, in their words, “better” opportunities. As lesbian, gay and bisexual people on the African continent, they emphasised that they moved to South Africa for the “protection” of being out and lesbian, gay, and bisexual that the Constitution and Bill of Rights promises. The poets indicated that they have specifically moved to Johannesburg, known locally as eGoli (city of gold); a reference to when gold was discovered in the reef upon which Johannesburg, initially a temporary mining camp, was established in the late 1800s. Known as a “city of migrants” (Crush, 2006), Johannesburg continues to attract migrants from elsewhere in South Africa and beyond who continue to search for today’s gold: improved livelihood opportunities. However, many migrants, both South African and non-national, and asylum seekers find limited access to resources and opportunities in the city (Landau, 2006; Landau, 2007; Vearey, 2010). In his essay ‘Migrants welcome,’ Nigerian-American writer, art historian, and photographer Teju Cole, says:

Some of the refugees become migrants, once the immediate danger is past. Some migrants become refugees, caught in an unexpected vortex of malice. Don’t let yourself be spun into a language of hatred and exclusion, at this hot moment in which it’s deemed OK to support refugees but still condemn migrants. I say refugee, I say migrant, I say neighbor, I say friend, because everyone is deserving of dignity. Because moving for economic benefit is itself a matter of life and death. Because money is the universal language, and to be deprived of it is to be deprived of a voice while everyone else is shouting. Sometimes the gun aimed at your head is grinding poverty, or endless shabby struggle, or soul crushing tedium (Cole, 2015).

When the poets in our workshop shared that they found their home towns or countries to be “unbearable” and they felt “uncared for” there they sought out Johannesburg as a safe haven. Although they experience Johannesburg as better, with “shackles broken” and “paths chosen,” as migrants they feel that their refuge is transitory or temporary and that they are constantly rebuilding a sense of home.

The end of Apartheid and the development of South Africa’s Constitution is associated with a so-called non-violent and “miraculous” transition (Klug, 2007: 83), but Apartheid created a lasting “ethos of violence” (Barbarin and Richter, 2013:93). As migrants in South Africa, the poets in our workshop often experience verbal and physical xenophobic violence (Landau, 2009), as they cross borders, seek visas, and/or go about their daily routine in Johannesburg. At times, they negotiate and navigate “acting straight” or “showing up strong” as they experience biopower (Foucault, 1978; Rose, 2006) in Johannesburg. Our participants are enacting an expanded version of biopower, what Creary terms “biocultural citizenship” (2016: 88), which is the complex interplay of biological, cultural, and societal factors among individuals who strive to gain full citizenship. Nevertheless, even if at times they feel safe, they believe that this safety is not guaranteed.

In ‘No serenity here,’ Kgositsile said:

In my language there is no word for *citizen*, which is an ingredient of that 19th century omelette. That word came to us as part of the package that contained the bible and the rifle. But moagi, resident, is there and it has nothing to do with any border or boundary you may or may not have crossed before waking up on the piece of earth where you currently live (Kgositsile, 2010:148).

Although “citizen” may not be found in indigenous South African languages, migrants to Johannesburg, from elsewhere in South Africa and beyond, still experience tenuous citizenship. LGB migrants and asylum seekers in South Africa navigate the spectrum between invisibility and hypervisibility in their home communities and countries, as well as in Johannesburg. In relation to their tribes, their institutions of faith, and the state, they often “shapeshift” (Cox, 2015) in their daily lives in order to resist dehumanisation. In an example of shapeshifting and reclamation, on the fifth and final day of the poetry workshop, the poets publically read and performed their poetry at Constitution Hill in Johannesburg, the site of a former prison complex where leaders of South African liberation groups were once detained, and the seat of the Constitutional Court of South Africa - the highest court in the country.

The poets begin to pursue freedom by migrating to the safe haven, however tenuous, of Johannesburg. They imagine freedom through writing and performing their poetry. They continue to pursue “freedom” being out as lesbian, gay, or bisexual, in their words “taking off masks” and “not hiding.” They conceive of freedom as being able to “chill” and as a “release.” Moreover, LGB migrants and asylum seekers work to imagine and pursue freedom in Johannesburg, creating what scholar-activist Ruth Wilson Gilmore calls “abolition geographies” (Gilmore, 2015). Through their participation in *Queer Crossings* since 2014, including in the #weareallpoets workshop in 2015 and the exhibition in 2016, LGB migrants and asylum seekers have built social trust or network-based social capital (Carpiano, 2007) in Johannesburg; this involves developing and leveraging social support mechanisms and networks. Over 60 years ago, South Africa’s Freedom Charter declared “that our country will never be prosperous or free until all people live in brotherhood, enjoying equal rights and opportunities” (African National Congress, 1955). The LGB migrants and asylum seekers that we worked with imagine the barriers to and possibilities of such freedom in South Africa though their poetry, and hope to pursue such freedom in their living.

## Notes

1. In this article, rather than using LGBTIQ or queer, we use lesbian, gay, and bisexual (LGB) to reflect the ways in which our participants in the *Queer Crossings* Project specifically refer to themselves.

2. In this article, we do not disclose the reported documentation status of project participants.

3. see: http://www.migration.org.za/move

4. All poems produced during the workshop, along with the biographies of the participating poets, can be found on the project blog: https://weareallpoets2015.wordpress.com

5. All names are pseudonyms, chosen by the poets.

6. Erasure Poetry is a poetic form of “found poetry” created by erasing words from an existing text, and then using the leftover words to write a poem. Some erasure poems work with or against the original text, while other erasure poems look for completely new and separate meanings than the original text.

7. see: https://issuu.com/move.methods.visual.explore/docs/queer_crossings_publication_v.2_-_f

8. A radio story was published about the event: https://radio.abc.net.au/programitem/pgobVwaL9G?play=true
